# Mental Health Impacts in Argentinean College Students During COVID-19 Quarantine

**DOI:** 10.3389/fpsyt.2021.557880

**Published:** 2021-03-04

**Authors:** Lorena Cecilia López Steinmetz, Candela Abigail Leyes, María Agustina Dutto Florio, Shao Bing Fong, Romina Lucrecia López Steinmetz, Juan Carlos Godoy

**Affiliations:** ^1^Laboratorio de Psicología, Instituto de Investigaciones Psicológicas (IIPsi), Facultad de Psicología, Universidad Nacional de Córdoba (UNC) - Consejo Nacional de Investigaciones Científicas y Técnicas (CONICET), Córdoba, Argentina; ^2^Decanato de Ciencias Sociales, Universidad Siglo 21, Córdoba, Argentina; ^3^University of Melbourne, Faculty of Science, Melbourne, VIC, Australia; ^4^Instituto de Evolución, Ecología Histórica y Ambiente (IDEVEA), Universidad Tecnológica Nacional (UTN) - Consejo Nacional de Investigaciones Científicas y Técnicas (CONICET), San Rafael, Mendoza, Argentina

**Keywords:** coronavirus disease (COVID-19), quarantine, anxiety, learned helplessness, social isolation, depressive symptoms, COVID-19

## Abstract

**Background:** We aimed to: (1) analyze differences in both general (in terms of psychological well-being/discomfort, social functioning and coping, and psychological distress) and specific (depression, trait-anxiety, negative alcohol-related consequences, and suicidal risk) mental health state (MHS) in college students, residing in four different Argentinean regions (center, north, south, and the most populated) exposed to different spread-rates of the COVID-19; (2) analyze between-group differences in both general and specific MHS indicators at four quarantine sub-periods (twice prior, and twice following the first quarantine extension).

**Methods:** We used a cross-sectional design with a convenience sample including 2,687 college students. Data was collected online during the Argentinean quarantine. We calculated one-way between-groups ANOVA with Tukey's *post hoc* test.

**Results:** Regionally, the center and the most populated area differed in psychological well-being/discomfort and negative alcohol-related consequences, but not in the remaining MHS indicators. According to the quarantine sub-periods, there were differences in psychological well-being/discomfort, social functioning and coping, psychological distress, and negative alcohol-related consequences. Negative alcohol-related consequences were the only MHS indicator improving over time. For all of the remaining MHS indicators, we found a similar deterioration pattern in the course of time, with mean scores decreasing from the first to the 2nd week of the quarantine pre-extensions, then increasing toward the 1st week of the quarantine post-extension (with some MHS indicators reaching mean scores worse than the start), and then continued to increase.

**Conclusion:** A worsened mean MHS during quarantine suggests that quarantine and its extensions contribute to negative mental health impacts.

## Introduction

Coronavirus disease (COVID-19) is an infectious disease caused by a newly discovered coronavirus. The current outbreak started in China during late 2019 and subsequently spread around the world. On 11th March 2020, the World Health Organization (WHO) declared this outbreak as a pandemic (1). Until when effective vaccines against COVID-19 are available on a large scale, social-distancing including travel bans, is one of the most effective interventions to contain the spread of the pandemic. Isolation and quarantine are the control and preventive measures most used by governments. While isolation consists of separating people who have been diagnosed with a contagious disease, from the general population, quarantine consists of separating and restricting the movement of people who are not sick, but may potentially been exposed to a contagious disease, thus reducing the risk of infecting others (2).

By the end of March 2020, a third of the world's population was living under quarantine (3). In Latin America, Argentina was one of the countries earliest in adopting varied social-distancing preventive interventions and related socio-economic decisions since 10th March 2020 (4). A presidential decree (number 297/2020) established that quarantine became mandatory for all Argentinean inhabitants—except for those working in essential services—from 20th to 31st March 2020. However, on 29th March, the first quarantine extension was announced for until 13th April. Then on 10th April, a second extension was implemented by the Government for until 26th April, and subsequently several additional extensions were implemented thereafter, reaching a quarantine duration of 285 days.

Reviews on the psychological impact of previous quarantine situations reported negative psychological effects related to quarantine, e.g., post-traumatic stress, depressive and anxiety symptoms, anger, distress, and other general psychological symptoms (5). Moreover, some of these quarantine effects would be long-lasting (6). As for the current COVID-19 pandemic, negative psychological impact including depression and anxiety symptoms have also been reported in China during the initial stage of this pandemic (7). Strikingly, in younger aged groups, there are contradictory findings suggesting both that quarantine does not have immediate negative psychological effects (e.g., in undergraduate students) (8) and that young people experience greater anxiety and depression compared to older people [Urquijo as cited in (9)].

Evidence is also not conclusive on pre-quarantine predictors of psychological impact, but a younger age (16–24 years) and the female gender were reported to be associated with such impacts (10). Having a history of psychiatric illness was associated with anxiety even several months after quarantine has ended (6). Stressors during quarantine included quarantine duration, fears of infection, frustration and boredom, inadequate supplies, and inadequate information (5). Notwithstanding, longer durations of quarantine (e.g., 10-day duration) (11) were reported to either result in higher negative psychological effects (11, 12) or having no significant effect (e.g., in anxiety levels) [Urquijo as cited in (9, 13)], and it was even suggested that a kind of accustoming would occur [Urquijo as cited in (9)]. In parallel, it was described that an extension of quarantine duration, irrespective of how small, is likely to exacerbate negative psychological effects (14).

Taken together, there is certitude that the current *world quarantine* was unprecedented and the psychological effects of quarantining a city, a country, or a third of the world, are unknown. However, regardless of whether it succeeds in controlling the pandemic, it is expected that the widespread quarantine will inevitably have a psychological effect (15). Equally, in Argentina, having the whole country population under quarantine was unprecedented and the subsequent psychological impacts are unknown. The effect of large-scale disease outbreaks on adolescents' mental health is an important gap for research (16). College closures substantially disrupt the lives of students (16, 17). In addition, the psychological impacts in Argentinean populations from different regions may differ among them due to two main reasons. One, they have different idiosyncratic features. Two, they were exposed to different spread-rates of the COVID-19 (18). The aims of this research are 2-fold: (1) to analyze differences in both general (i.e., in terms of psychological well-being/discomfort, social functioning and coping, and psychological distress) and specific (i.e., in terms of depression, trait-anxiety, negative alcohol-related consequences, and suicidal risk) mental health state (MHS) in college students, residing in provinces from four different regions (north, center, south, and the most populated) of Argentina exposed to different spread-rates of the COVID-19; (2) to analyze between-group differences in both general and specific MHS indicators at four quarantine sub-periods (twice prior, and twice following the first quarantine extension).

## Method

### Sample and Procedure

This study used a cross-sectional design. Sampling was one of convenience. Data were collected since 17th March (i.e., 3 days before quarantine became mandatory, but when quarantine was already strongly recommended by the Government to all Argentinean inhabitants) until 29th April 2020 (i.e., during the mandatory Argentinean quarantine). Collection procedure was carried out *via* online, by using the LimeSurvey software (UNC license). For data collection, this study was posted many times on social networks (Facebook, Twitter, and Instagram) and then liked, re-tweeted, and/or shared by many people, throughout the period of Argentina's quarantine analyzed in this study. The invitations to participate contained a brief mention to the general aim, inclusion criteria (being a college student at any public or private university in Argentina, being Argentinean, having 18 years of age or older, currently residing in one of the following Argentinean provinces: Jujuy, Salta, Santa Cruz, Tierra del Fuego, Córdoba or Buenos Aires), and the link for the online survey. Upon accessing the survey, participants were initially presented with the information sheet and informed consent form approved by the Ethics Committee of the Institute of Psychological Research, Faculty of Psychology, National University of Córdoba. After giving their consent to participate, participants were presented with a series of questions aimed to check compliance with the inclusion criteria. Safety procedures included a feedback email to each subject after participation, which contains the scores obtained in each instrument along with a brief description on what these scores mean, and contact information on mental health services available free of charge. These emails also had the function to raise awareness of their own-mental health status.

**Figure 1:**
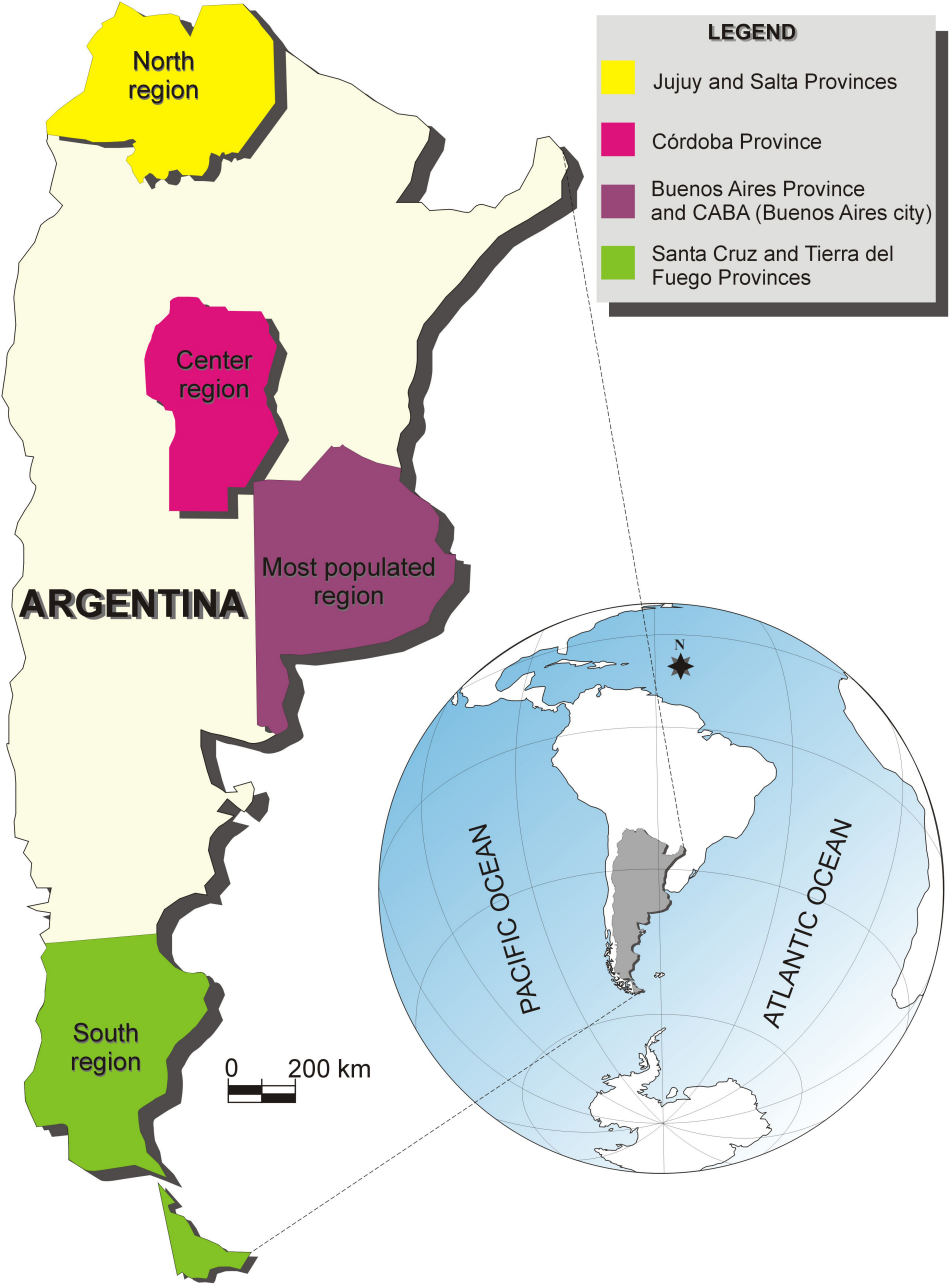
Map of Argentina showing the location of the northern, central, most populated, and southern regions. The most populated area corresponds to the Buenos Aires City (CABA) and the Buenos Aires Province.

A total of 3,870 Argentinean college students participated in the online survey, but 1,183 (30.57%) did not complete the survey. In this paper, we focused only on the sample that completed the online survey. Therefore, the sample was composed of 2,687 college students (81.58% women, 17.60% men, 0.82% other) from 18 years of age (M_age_ = 22.74, standard deviation [±SD] ±3.64), residing in one of six different Argentinean provinces (Figure 1).

### Instruments

(A) *General Mental Health State (GMHS)*

*Psychological well-being/discomfort* and *Social functioning and coping*. We used the General Health Questionnaire (GHQ-12) (19), in its Argentinean validation (Cronbach's alpha = 0.80) (20). This is a 12-item measure, which evaluates the general dimension of self-perceived health and allows for discrimination in two dimensions (six items each): (a) unspecific psychological well-being/discomfort, and (b) social functioning and coping. The higher the score, the worse is the self-perceived health.

*Psychological distress*. We used the Kessler Psychological Distress Scale (K-10) (21), in its Argentinean validation (Cronbach's alpha = 0.88) (22). This is a 10-item global dimensional measure of non-specific psychological distress, which evaluates symptoms related to depression and anxiety, indicating the risk to suffer psychological distress but does not specify the disorder. Higher scores indicate higher psychological distress.

(B) *Specific Mental Health State (SMHS)*

*Depression*. We used the Beck Depression Inventory (BDI-II) (23) in its Argentinean version (Cronbach's alpha = 0.86) (24). This is a 21-item instrument measuring depression and its severity. Its items describe the most frequent clinical symptoms of depressed subjects. In non-clinical populations, scores above 20 indicate depression (25).

*Trait-Anxiety*. We used the 20-items subscale for trait-anxiety of the State-Trait Anxiety Inventory (STAI) in its Spanish version (Cronbach's alpha = 0.84/0.87) (26). This subscale measures anxiety-related symptoms, such as restlessness, nervousness, and agitation. Higher scores indicate more anxiety symptoms.

*Negative alcohol-related consequences*. We used the Brief Young Adult Alcohol Consequences Questionnaire (B-YAACQ) (27), in its Argentinean version (Cronbach's alpha = 0.78) (28). This is a 24-item measure on negative alcohol-related consequences over the past year among college students. Higher scores indicate worse alcohol-related consequences.

*Suicidal risk*. We used the Inventory of Suicide Orientation (ISO-30) (29), in its Argentinean validation (Cronbach's alpha = 0.88) (30), a 30-item evaluation tool which helps in identifying suicidal risk. Higher scores indicate higher suicidal risk.

### Data Analysis

We performed all data analysis with RStudio version 3.6.3 (31). We considered *p*-values ≤ 0.05 as statistically significant. We reported exact *p*-values, except for *p*-values under 0.001, where we reported as < 0.001. Likewise, 95% confidence intervals (CI) were informed when corresponded. Skewness and kurtosis were calculated in all factors of both general and specific MHS. Since these scores were in the range of acceptable values or near to (−3 and 3) (32), parametric tests were applied. Given that during data collection all items were marked as mandatory response, there were no missing data to handle. For addressing the two aims of this research, we applied one-way between-groups ANOVA with Tukey's *post hoc* test.

For analyses corresponding to the first aim, we divided the entire sample into four groups: (a) participants residing in Jujuy and Salta provinces, named as the *north* region (*n* = 371); (b) participants residing in Córdoba province, named as the *center* region (*n* = 1,048); (c) participants residing in Santa Cruz and Tierra del Fuego provinces, named as the *south* region (*n* = 89); (d) participants residing in Buenos Aires [including both the Buenos Aires City (CABA) and the Buenos Aires Province], named as the *most populated* region (*n* = 1,179).

For analyses corresponding to the second aim, we divided the entire sample into four groups: (a) participants answering during 17–23 March 2020, i.e., 1st week of data collection before the quarantine extension, named as *1st week of quarantine pre-extension* (*n* = 1508); (b) participants answering during 24–29 March 2020, named as *2nd week of quarantine pre-extension* (*n* = 525); (c) participants answering during 30 March to 05 April 2020, named as *1st week of quarantine post-extension* (*n* = 364); (d) participants answering during 06–29 April 2020, named as *remaining weeks of quarantine post-extension* (*n* = 290).

## Results

### Differences in Mental Health State by Regions

Regarding general MHS by regions, a statistically significant difference was found in psychological well-being/discomfort [*F*_(3)_ = 4.57, *p-value* = 0.003]. This difference was observed between the center and the most populated region, but not between the remaining regions (Table 1). Mean scores (±SD) of psychological well-being/discomfort were (in decreasing order) 3.21 (±1.83) in the most populated region, 3.15 (±1.84) in the north, 3.10 (±1.73) in the south, and 2.92 (±1.84) in the center. Conversely, no significant differences by regions were found in social functioning and coping [*F*_(3)_ = 1.51, *p-value* = 0.21] (Table 1), with mean scores of 2.26 (±1.96) in the north, 2.19 (±1.90) in the most populated region, 2.18 (±1.87) in the south, and 2.06 (±1.83) in the center. Likewise, no significant differences by regions were found in psychological distress [*F*_(3)_ = 1.31, *p-value* = 0.27] (Table 1), with mean scores of 26.30 (±7.80) in the south, 25.76 (±8.09) in the most populated region, 25.46 (±8.20) in the north, and 25.16 (±8.12) in the center.

**Table 1:** Multiple comparisons^a^ of means in mental health state scores by regions.

**Regions**	**Dif**	**Lower**	**Upper**	***p* adj**
**Psychological well-being/discomfort**				
Most populated–Center	0.28	0.08	0.48	0.002
North–Center	0.23	−0.05	0.51	0.16
South–Center	0.18	−0.34	0.70	0.82
North–Most populated	−0.05	−0.33	0.23	0.96
South–Most populated	−0.10	0.62	0.41	0.95
South–North	−0.05	−0.61	0.50	0.99
**Social functioning and coping**				
Most populated–Center	0.14	−0.07	0.34	0.31
North–Center	0.20	−0.09	0.49	0.28
South–Center	0.12	−0.41	0.66	0.93
North–Most populated	0.06	−0.22	0.35	0.94
South–Most populated	−0.01	−0.55	0.52	0.99
South–North	−0.08	−0.65	0.49	0.98
**Psychological distress**				
Most populated–Center	0.60	−0.28	1.48	0.30
North–Center	0.30	−0.96	1.56	0.93
South–Center	1.14	−1.16	3.44	0.58
North–Most populated	−0.30	−1.54	0.94	0.92
South–Most populated	0.54	−1.75	2.83	0.93
South–North	0.84	−1.62	3.30	0.81
**Depression**				
Most populated–Center	0.86	−0.34	2.06	0.25
North–Center	1.21	−0.50	2.92	0.26
South–Center	1.69	−1.43	4.81	0.50
North–Most populated	0.35	−1.33	2.03	0.95
South–Most populated	0.83	−2.28	3.93	0.90
South–North	0.48	−2.86	3.81	0.98
**Anxiety**				
Most populated–Center	0.69	−0.55	1.94	0.47
North–Center	0.63	−1.14	2.39	0.80
South–Center	1.90	−1.33	5.13	0.43
North–Most populated	−0.07	−1.81	1.67	0.99
South–Most populated	1.20	−2.01	4.42	0.77
South–North	1.27	−2.18	4.72	0.78
**Negative alcohol-related consequences**				
Most populated–Center	−0.69	−1.11	−0.27	0.0002
North–Center	−0.19	−0.79	0.40	0.84
South–Center	0.31	−0.78	1.40	0.89
North–Most populated	0.49	−0.10	1.08	0.14
South–Most populated	0.99	−0.09	2.08	0.09
South–North	0.50	−0.66	1.67	0.68
**Suicidal risk**				
Most populated–Center	1.27	−0.51	3.06	0.26
North–Center	1.34	−1.20	3.88	0.53
South–Center	2.90	−1.74	7.54	0.38
North–Most populated	0.07	−2.44	2.57	0.99
South–Most populated	1.63	−3.00	6.25	0.80
South–North	1.56	−3.41	6.52	0.85

*Dif, Difference; Lower–Upper, Lower and upper limits of 95% confidence intervals; p adj, Adjusted p-value; North, Jujuy and Salta provinces; Center, Córdoba province; South, Santa Cruz and Tierra del Fuego provinces; Most populated, City of Buenos Aires (Ciudad Autónoma de Buenos Aires, CABA) and the Buenos Aires Province*.

a *Multiple comparison of means based on Tukey's post hoc test*.

Regarding specific MHS by regions, a statistically significant difference was found in negative alcohol-related consequences [*F*_(3)_ = 6.90, *p-value* < 0.001]. This difference was observed between the most populated and the center region, but not between the remaining regions (Table 1). Mean scores were 4.33 (±4.23) in the south, 4.02 (±3.88) in the center, 3.82 (±4.21) in the north, and 3.33 (±3.66) in the most populated region. Conversely, no significant differences by regions were found in depression [*F*_(3)_ = 1.94, *p-value* = 0.12], anxiety [*F*_(3)_ = 1.24, *p-value* = 0.29], nor in suicidal risk [*F*_(3)_ = 1.78, *p-value* = 0.15] (Table 1). In depression, mean scores were 19.09 (±10.76) in the south, 18.61 (±11.00) in the north, 18.26 (±11.16) in the most populated region, and 17.40 (±10.83) in the center. In anxiety, mean scores were 30.68 (±10.27) in the south, 29.48 (±11.57) in the most populated region, 29.41 (±11.09) in the north, and 28.79 (±11.35) in the center. In suicidal risk, mean scores were 36.62 (±15.92) in the south, 35.06 (±15.47) in the north, 34.99 (±16.60) in the most populated region, and 33.72 (±16.45) in the center.

### Differences in Mental Health State by Quarantine Sub-Periods

Regarding general MHS by quarantine sub-periods, statistically significant differences were found in psychological well-being/discomfort [*F*_(3)_ = 8.31, *p-value* < 0.001], in social functioning and coping [*F*_(3)_ = 8.14, *p-value* < 0.001], and in psychological distress [*F*_(3)_ = 3.65, *p-value* = 0.01]. These differences were observed between several quarantine sub-periods (Table 2). In psychological well-being/discomfort, social functioning and coping, and psychological distress, mean scores decreased from the 1st to the 2nd week of quarantine pre-extension, followed by an increase during the 1st week of quarantine post-extension (where mean scores were higher than the initial measurements in psychological well-being/discomfort and in social functioning and coping), and continued to increase in the remaining weeks of quarantine post-extension (Table 3; Supplementary Figures 1–3).

**Table 2:** Multiple comparisons^a^ of means in mental health state scores by quarantine sub-periods.

**Quarantine sub-periods**	**Dif**	**Lower**	**Upper**	***p* adj**
**Psychological well-being/discomfort**				
1.1st week of quarantine pre-extension−2. 2nd week of quarantine pre-extension	−0.14	−0.37	0.10	0.46
1.1st week of quarantine pre-extension−3. 1st week of quarantine post-extension	0.20	−0.07	0.48	0.23
1.1st week of quarantine pre-extension−4. Remaining weeks of quarantine post-extension	0.48	0.18	0.78	0.0003
2.2nd week of quarantine pre-extension−3. 1st week of quarantine post-extension	0.34	0.02	0.66	0.03
2.2nd week of quarantine pre-extension−4. Remaining weeks of quarantine post-extension	0.61	0.27	0.96	0.00003
3.1st week of quarantine post-extension−4. Remaining weeks of quarantine post-extension	0.27	−0.10	0.64	0.23
**Social functioning and coping**				
1.1st week of quarantine pre-extension−2. 2nd week of quarantine pre-extension	−0.03	−0.27	0.22	0.99
1.1st week of quarantine pre-extension−3. 1st week of quarantine post-extension	0.30	0.02	0.58	0.03
1.1st week of quarantine pre-extension−4. Remaining weeks of quarantine post-extension	0.50	0.19	0.81	0.0002
2.2nd week of quarantine pre-extension−3. 1st week of quarantine post-extension	0.33	0.002	0.66	0.05
2.2nd week of quarantine pre-extension−4. Remaining weeks of quarantine post-extension	0.53	0.18	0.88	0.0006
3.1st week of quarantine post-extension−4. Remaining weeks of quarantine post-extension	0.20	−0.18	0.58	0.53
**Psychological distress**				
1.1st week of quarantine pre-extension−2. 2nd week of quarantine pre-extension	−0.53	−1.58	0.53	0.57
1.1st week of quarantine pre-extension−3. 1st week of quarantine post-extension	−0.20	−1.42	1.01	0.97
1.1st week of quarantine pre-extension−4. Remaining weeks of quarantine post-extension	1.39	0.05	2.72	0.04
2.2nd week of quarantine pre-extension−3. 1st week of quarantine post-extension	0.32	−1.09	1.74	0.94
2.2nd week of quarantine pre-extension−4. Remaining weeks of quarantine post-extension	1.91	0.39	3.44	0.007
3.1st week of quarantine post-extension−4. Remaining weeks of quarantine post-extension	1.59	−0.05	3.23	0.06
**Depression**				
1.1st week of quarantine pre-extension−2. 2nd week of quarantine pre-extension	−0.52	−1.95	0.91	0.79
1.1st week of quarantine pre-extension−3. 1st week of quarantine post-extension	0.17	−1.48	1.83	0.99
1.1st week of quarantine pre-extension−4. Remaining weeks of quarantine post-extension	0.91	−0.90	2.73	0.56
2.2nd week of quarantine pre-extension−3. 1st week of quarantine post-extension	0.70	−1.23	2.62	0.79
2.2nd week of quarantine pre-extension−4. Remaining weeks of quarantine post-extension	1.44	−0.63	3.50	0.28
3.1st week of quarantine post-extension−4. Remaining weeks of quarantine post-extension	0.74	−1.49	2.97	0.83
**Anxiety**				
1.1st week of quarantine pre-extension−2. 2nd week of quarantine pre-extension	−0.83	−2.31	0.66	0.48
1.1st week of quarantine pre-extension−3. 1st week of quarantine post-extension	−0.50	−2.20	1.21	0.88
1.1st week of quarantine pre-extension−4. Remaining weeks of quarantine post-extension	0.51	−1.37	2.38	0.90
2.2nd week of quarantine pre-extension−3. 1st week of quarantine post-extension	0.33	−1.66	2.32	0.97
2.2nd week of quarantine pre-extension−4. Remaining weeks of quarantine post-extension	1.33	−0.81	3.48	0.38
3.1st week of quarantine post-extension−4. Remaining weeks of quarantine post-extension	1.00	−1.30	3.31	0.68
**Negative alcohol-related consequences**				
1.1st week of quarantine pre-extension−2. 2nd week of quarantine pre-extension	−0.30	−0.80	0.20	0.42
1.1st week of quarantine pre-extension−3. 1st week of quarantine post-extension	−0.38	−0.96	0.20	0.32
1.1st week of quarantine pre-extension−4. Remaining weeks of quarantine post-extension	−0.62	−1.26	0.01	0.05
2.2nd week of quarantine pre-extension−3. 1st week of quarantine post-extension	−0.08	−0.76	0.59	0.99
2.2nd week of quarantine pre-extension−4. Remaining weeks of quarantine post-extension	−0.32	−1.05	0.40	0.66
3.1st week of quarantine post-extension−4. Remaining weeks of quarantine post-extension	−0.24	−1.02	0.54	0.86
**Suicidal risk**				
1.1st week of quarantine pre-extension−2. 2nd week of quarantine pre-extension	−2.09	−4.22	0.04	0.06
1.1st week of quarantine pre-extension−3. 1st week of quarantine post-extension	−0.37	−2.82	2.09	0.98
1.1st week of quarantine pre-extension−4. Remaining weeks of quarantine post-extension	0.62	−2.08	3.31	0.94
2.2nd week of quarantine pre-extension−3. 1st week of quarantine post-extension	1.72	−1.15	4.58	0.41
2.2nd week of quarantine pre-extension−4. Remaining weeks of quarantine post-extension	2.70	−0.37	5.78	0.11
3.1st week of quarantine post-extension−4. Remaining weeks of quarantine post-extension	0.99	−2.32	4.30	0.87

*Dif, Difference; Lower–Upper, Lower and upper limits of 95% confidence intervals; p adj, Adjusted p-value; 1. 1st week of quarantine pre-extension, participants answering during 17–23 March 2020, i.e., 1st week of data collection before quarantine extension; 2. 2nd week of quarantine pre-extension, participants answering during 24–29 March 2020, i.e., 2nd week of data collection before quarantine extension; 3. 1st week of quarantine post-extension, participants answering during 30 March to 05 April 2020, i.e., 1st week of data collection after quarantine extension; 4. Remaining weeks of quarantine post-extension, participants answering during 06–29 April 2020, remaining weeks of data collection after quarantine extension*.

a *Multiple comparison of means based on Tukey's post hoc test*.

**Table 3:** Central tendencies and variability measures in mental health state scores by quarantine sub-periods.

**Mental health state indicators**	**Mean (±SD)**			
	**1st week pre-ext**.	**2nd week pre-ext**.	**1st week post-ext**.	**Rem. weeks post-ext**.
Psychological well-being/discomfort	3.03 (±1.82)	2.90 (±1.82)	3.24 (±1.89)	3.51 (±1.80)
Social functioning and coping	2.06 (±1.84)	2.03 (±1.83)	2.36 (±1.97)	2.56 (±1.99)
Psychological distress	25.48 (±7.98)	24.96 (±8.22)	25.28 (±8.00)	26.87 (±8.57)
Depression	17.98 (±10.91)	17.46 (±10.90)	18.16 (±10.80)	18.90 (±11.88)
Anxiety	29.42 (±11.23)	28.59 (±11.58)	28.92 (±11.25)	29.92 (±11.94)
Negative alcohol-related consequences	3.88 (±3.85)	3.58 (±3.73)	3.49 (±4.00)	3.25 (±3.93)
Suicidal risk	34.95 (±16.42)	32.86 (±16.04)	34.58 (±16.11)	35.56 (±16.87)

*±SD, standard deviation; 1st week-pre., 1st week of quarantine pre-extension (participants answering during 17–23 March 2020, i.e., 1st week of data collection before quarantine extension); 2nd week-pre., 2nd week of quarantine pre-extension (participants answering during 24–29 March 2020, i.e., 2nd week of data collection before quarantine extension); 1st week post-ext., 1st week of quarantine post-extension (participants answering during 30 March to 05 April 2020, i.e., 1st week of data collection after quarantine extension); Rem. weeks post-ext., Remaining weeks of quarantine post-extension (participants answering during 06–29 April 2020, remaining weeks of data collection after quarantine extension)*.

Regarding specific MHS by quarantine sub-periods, a statistically significant difference was found in negative alcohol-related consequences [*F*_(3)_ = 2.86, *p-value* = 0.03]. This difference was observed between the 1st week of quarantine pre-extension and the remaining weeks of quarantine post-extension, but not between the other sub-periods (Table 2). Mean scores of negative alcohol-related consequences decreased as quarantine sub-periods progressed (Table 3; Supplementary Figure 6). Conversely, no significant differences by quarantine sub-periods were found in depression [*F*_(3)_ = 1.09, *p-value* = 0.35], anxiety [*F*_(3)_ = 1.14, *p-value* = 0.33], nor in suicidal risk [*F*_(3)_ = 2.53, *p-value* = 0.055] (Table 2). In depression, anxiety, and suicidal risk, mean scores decreased from the 1st to the 2nd week of quarantine pre-extension, followed by an increase during the 1st week of quarantine post-extension (where mean scores were higher than the initial measurements in depression), and continued to increase in the remaining weeks of quarantine post-extension (Table 3; Supplementary Figures 4, 5, 7).

## Discussion

Toward the end of April, available official data (18) indicates that spread-rates of the COVID-19 were high in provinces such as Buenos Aires, were relatively high in center provinces (e.g., Córdoba), were medium in southern provinces (e.g., Tierra del Fuego), and were low in northern provinces (e.g., Jujuy and Salta). Our findings indicate that worse self-perceived health, in terms of unspecific psychological discomfort, affected more college students residing in the region with the highest COVID-19 spread-rates (i.e., most populated region), compared to those residing in the center region, where spread-rates are relatively high. Conversely, negative alcohol-related consequences affected less college students in the former region as compared to the latter.

On the other hand, living in regions with higher, medium or lower spread-rates of the COVID-19 do not appear to produce significant differences in social functioning and coping, psychological distress, depression, anxiety, nor suicidal risk. This would imply that such mental health impacts during quarantine may be attributed to aspects related to social distancing, isolation, and routine disruptions, rather than the objective risk of contagion.

Based on the literature, a negative psychological impact of quarantine was expected to be found (5, 15). Our findings confirmed this expectation, with additional insights upon duration, a relevant aspect in the impact of quarantine. Our findings indicated that, for people already in quarantine, an extension of quarantine duration exacerbated negative mental health impacts, escalating a sustained worsening on MHS as time went by. Therefore, our findings support the assertion that indefinite quarantine duration may be more detrimental on mental health than applying limited periods (5).

Negative alcohol-related consequences was the only MHS indicator that improved over time, suggesting that higher alcohol consumption among college students is dependent on contexts of consumption (33, 34) and positive alcohol expectancies (33, 35, 36). Except for negative alcohol-related consequences, our findings revealed a similar worsening pattern for all the remaining MHS indicators as time went by. This pattern consisted in mean scores decreasing from the 1st to the 2nd week of quarantine pre-extension, then increasing toward the 1st week of quarantine post-extension (with some MHS indicators reaching mean scores worse than initially measured), and continued to increase thereon.

We disagree with the viewpoint that enquiring on suicidal thoughts or behaviors during quarantine may be “counterproductive” and, thus, should be avoided [Urquijo as cited in (9, 37)]. This kind of viewpoint both in research and clinical settings creates a catch-22 situation (38). Contrary to this, we based our standpoint from the available literature indicating that, by asking and talking about suicide may in fact reduce, rather than increase, suicidal ideation and may lead to improvements in mental health in treatment-seeking populations (38, 39). For these reasons, in this study we have administered a specific instrument for measuring suicidal risk, which demonstrated that suicidal risk follows the same worsening pattern as the other MHS indicators.

There are opposing findings on whether quarantine does [Urquijo and Andrés as cited in (9)] or does not (8) cause negative psychological effects in young people. Conspicuously, different studies presented a similar argument based on typical behaviors, customs, and responsibilities of young people in order to interpret these divergent findings. Indeed, it was suggested that quarantine does not cause negative mental health effects in young people, such as undergraduate students, as they have fewer responsibilities than adults who are employed full-time (5). Similarly, it was argued that young people currently under quarantine would experience the highest levels of anxiety and depression as they are accustomed to socialization and to have more community relationships outside of their homes than adults [Urquijo as cited in (9)]. While it is tacitly assumed that young people have fewer liabilities and/or responsibilities than adults, young people—for instance, college students—have liabilities and responsibilities related to their studies and, in many cases, also related to their parallel employments. Likewise, such interpretation does not comment on the influence of relevant factors, such as significantly reduced face-to-face social interactions, limited outdoor opportunities, living space adequacy (e.g., size, brightness, and privacy), disruption of routine activities, and experiences and attitudes toward COVID-19, among others, acting upon young people during quarantine. These latter factors are postulated to have more relevance, than the amount of responsibilities, in the interpretation of psychological impacts of quarantines (40–42). Concerning routines aforementioned, Urquijo [as cited in (9)] as well as Canet Juric et al. (37) suggested that current depressive and anxiety symptoms, and negative emotions decrease in the Argentinean population as time passes, by reason of accustoming to the quarantine. However, our observations are not in-line with this assertion and thus, we propose an alternative hypothesis for interpreting such findings. In this regard, we hypothesize that subjective perceptions of symptoms may have changed gradually, perhaps mimicking a passage from egodystonic to egosyntonic perception—which can be confounded with a health improvement or a positive adaptive behavior—although, as it is known, egosyntonic is not always a synonym of health [see, e.g., (43)]. As a result, self-reported scores on anxiety decreased [Urquijo and Andrés as cited in (9, 37)], but for a different reason from what was argued by these authors. We think that such a decrease does not imply that isolation or quarantine may be natural for human beings or, in other words, that people become accustomed to this situation. During quarantine, alike other situations (e.g., marital violence), people may tend to accept or naturalize situations, behaviors or reactions that are abnormal or unhealthy, but it is the role of healthcare workers and scientists to warn about these processes rather than legitimize it. Indeed, we propose that such a decrease in self-reported scores on anxiety and the increase in scores on depression [Urquijo as cited in (9, 37)] are more likely caused by a state of learned helplessness instead of a positive adaptive “accustoming” as stated by these authors.

Regarding the learned helplessness paradigm, this has long been proven to be a valid and reliable depression-like behavior model in animals (44) and has been shown to be reproducible in human subjects (45). The developmental trajectory described in animal models as learned helplessness or social defeat consists, in brief: 1°) the organism exhibits increasing anxiety-like behaviors, searching for ways of escaping or controlling an environment that has become threatening, 2°) the organism generalizes the learning that he/she has no control over its environment and anxiety-like behaviors decrease, 3°) further generalizes that the environment is inherently threatening and depression develops or increases, and 4°) ultimately leads the organism to give up (46). Our findings—and to some extent, results reported by Urquijo and Andrés [as cited in (9)], and Canet Juric et al. (37)—may correspond, point-by-point, with this developmental trajectory: steps 1° and 2° of this trajectory would correspond to our results during the first and second period of the quarantine pre-extension, e.g., with anxiety decreasing from the 1st to the 2nd week; step 3° of this trajectory would correspond with the worsening in MHS indicators (1st week of quarantine post-extension), e.g., mean scores on depression worsen than at the start and then approaching clinical depression; and step 4° of this trajectory would be represented in our results by the increased deterioration in MHS indicators (remaining weeks of quarantine post-extension). The effects of learned helplessness have a strong impact not only on behavior but also on physiological functioning, e.g., producing stress-induced analgesia and the activation of endogenous opiate systems (47). Fortunately, these effects can be reversed, for instance, by antidepressant treatment (48, 49), therapy (50), and also *via* experiencing controllable events (51). Evidently, in order for treatments and prevention to be possible, we need to be familiar with these processes rather than simply assuming that people naturally become accustomed to being quarantined.

Findings of our study may be useful for public health officials and government officials who must decide upon sanitary measures, public policies, and communication; however, they need to be interpreted with caution and considered within the context of several limitations. First, this study was cross-sectional, and prospective research is warranted to test hypotheses emerged from here. Second, our sample was one of convenience and it is unclear to what extent our results could be representative of the Argentinean population. However, we have used a sample as representative as possible, by including participants from different Argentinean regions, each one representing different idiosyncratic features and exposed to different spread-rates of the COVID-19. Third, this study has focused on university students, which could differ from young people not in the university (52), but who are also quarantined. Fourth, along with the quarantine and its extensions, additional factors not assessed in this study, such as fear of COVID-19 infection, pre-existing vulnerabilities, and financial consequences, among others, could have influence on the mental health outcomes. Despite these limitations, we think that our findings remain valuable and help shed light for further research on mental health impacts of the current quarantine, which is a pressing public health concern.

## Data Availability Statement

The data that support the findings of this study and the reproducible R code for data analysis are available in the Open Science Framework (OSF) repository, doi: 10.17605/OSF.IO/ZRX6T.

## Ethics Statement

The studies involving human participants were reviewed and approved by Comité de Ética del Instituto de Investigaciones Psicológicas de la Facultad de Psicología de la Universidad Nacional de Córdoba y del CONICET. The patients/participants provided their written informed consent to participate in this study.

## Author Contributions

LL has elaborated the research project, designed the online protocol for this research, participated in the data collection, has written the R code, performed the data analysis, and written the manuscript. CL and MD have participated in the data collection and carried-out bibliography searches. SF has participated in the data collection, made bibliography searches, and revised the manuscript for English grammar. RS has participated in the data collection, made bibliography searches, elaborated the Figure 1, and revised the manuscript. JG has participated in the data collection, made bibliography searches, supervised the study, and revised the manuscript. All authors contributed to the article and approved the submitted version.

## Conflict of Interest

The authors declare that the research was conducted in the absence of any commercial or financial relationships that could be construed as a potential conflict of interest.
