# Supplementary material for: Mental Health Impacts in Argentinean College Students During COVID-19 Quarantine
Source: Front Psychiatry. 2021 Mar 4;12:557880. doi: 10.3389/fpsyt.2021.557880 (PMC7969711; doi:10.3389/fpsyt.2021.557880)
Supplement: Supplementary file 1 [file Data_Sheet_1.docx]

**Supplementary materials for:**

***Mental health impacts in Argentinean college students during COVID-19 quarantine***

**INDEX**

| **Figure S1.** Psychological well-being/discomfort by quarantine sub-periods. Mean plot with 95% Confidence Interval. |  | **2** |
| --- | --- | --- |
| **Figure S2.** Social functioning and coping by quarantine sub-periods. Mean plot with 95% Confidence Interval. |  | **3** |
| **Figure S3.** Psychological distress by quarantine sub-periods. Mean plot with 95% Confidence Interval. |  | **4** |
| **Figure S4.** Depression by quarantine sub-periods. Mean plot with 95% Confidence Interval. |  | **5** |
| **Figure S5.** Anxiety by quarantine sub-periods. Mean plot with 95% Confidence Interval. |  | **6** |
| **Figure S6.** Negative alcohol-related consequences by quarantine sub-periods. Mean plot with 95% Confidence Interval. |  | **7** |
| **Figure S7.** Suicidal risk by quarantine sub-periods. Mean plot with 95% Confidence Interval. |  | **8** |

**
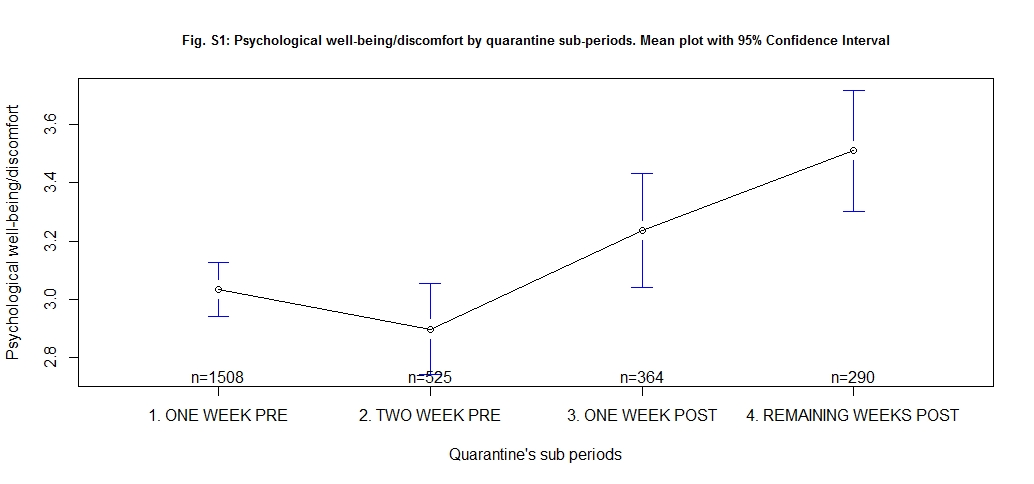
**

**Figure S1.** Psychological well-being/discomfort by quarantine sub-periods. Mean plot with 95% Confidence Interval.


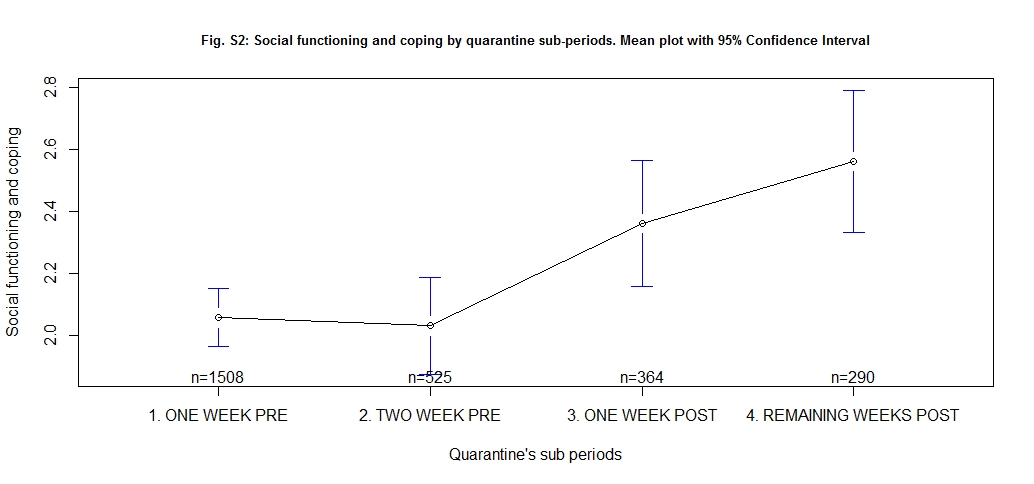


**Figure S2.** Social functioning and coping by quarantine sub-periods. Mean plot with 95% Confidence Interval.


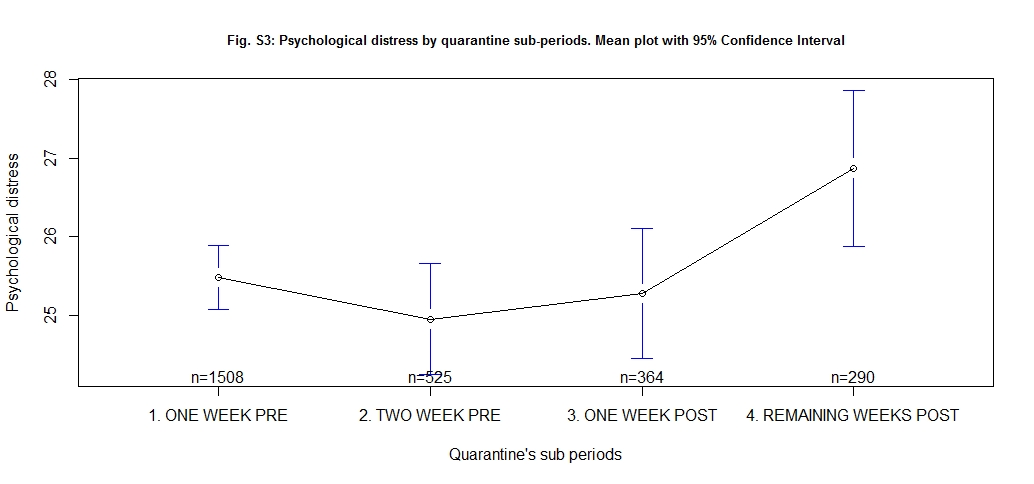


**Figure S3.** Psychological distress by quarantine sub-periods. Mean plot with 95% Confidence Interval.


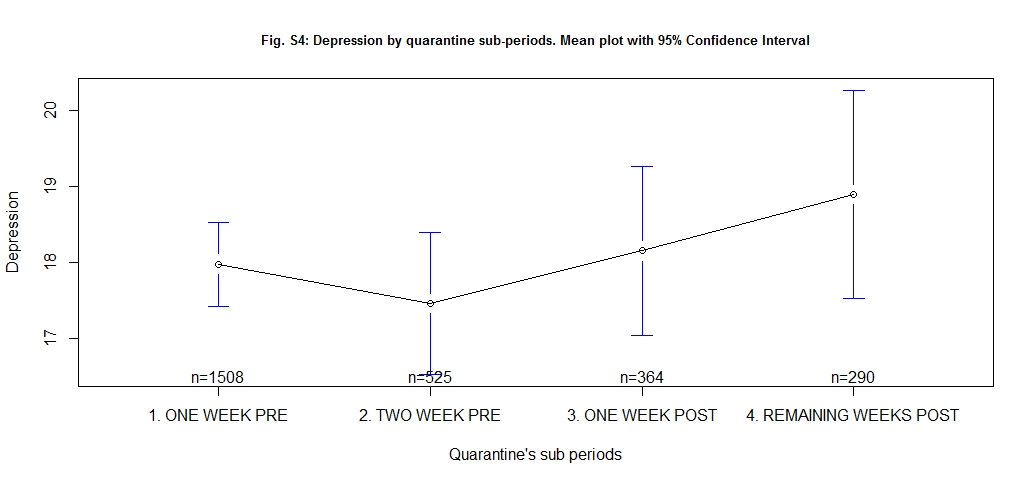


**Figure S4.** Depression by quarantine sub-periods. Mean plot with 95% Confidence Interval.


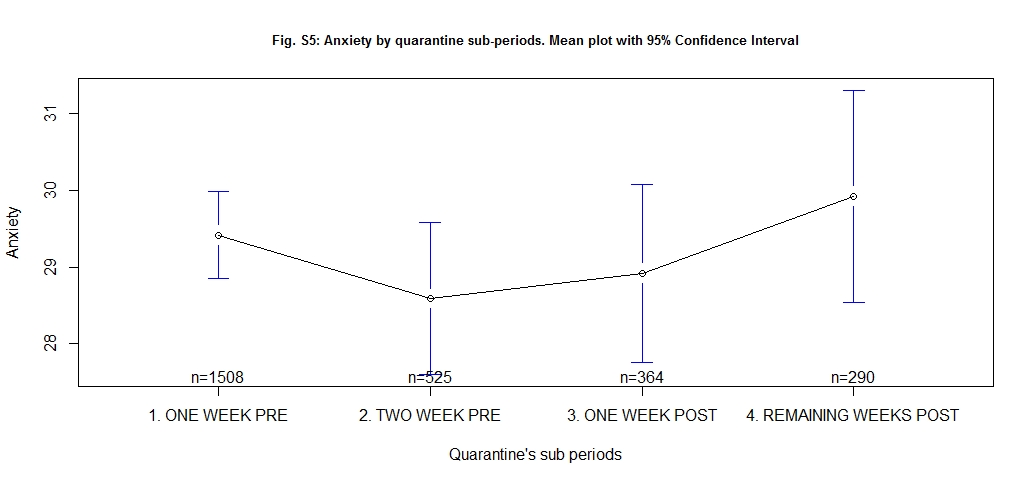


**Figure S5.** Anxiety by quarantine sub-periods. Mean plot with 95% Confidence Interval.


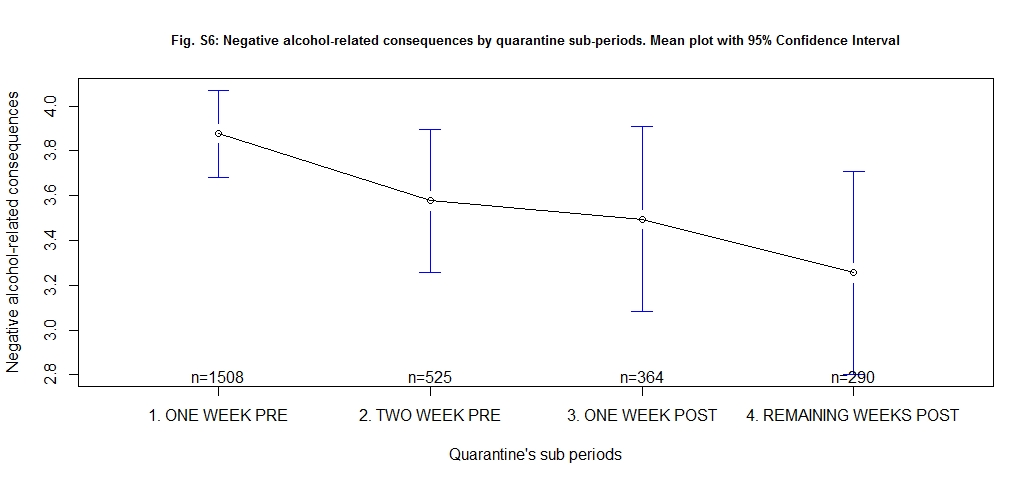


**Figure S6.** Negative alcohol-related consequences by quarantine sub-periods. Mean plot with 95% Confidence Interval.


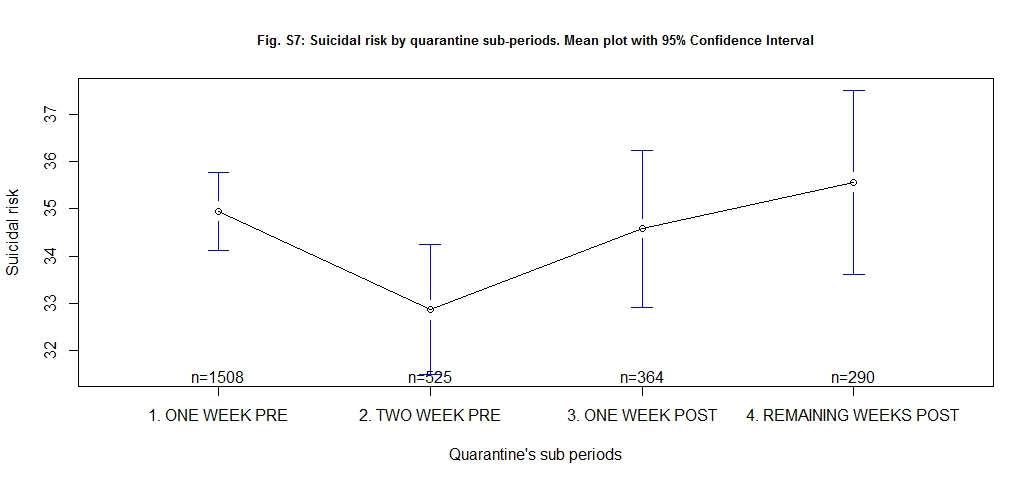


**Figure S7.** Suicidal risk by quarantine sub-periods. Mean plot with 95% Confidence Interval.
